# Supplementary material for: Genome co-adaptation and the evolution of methicillin resistant Staphylococcus aureus
Source: Genome Biol. 2026 Feb 11;27:91. doi: 10.1186/s13059-026-04000-6 (PMC12997946; doi:10.1186/s13059-026-04000-6)
Supplement: Supplementary file 1 — Additional file 1: Supplementary Figures S1, S2, S3. [file 13059_2026_4000_MOESM1_ESM.docx]

**Fig. S1: Site-specific integration of SCCmec**. (A) The S. aureus genome before and after SCCmec insertion. The attB (blue) and attS (red) sites are responsible for SCCmec recombination. Before insertion, the attB site is located downstream of the rlmH sequence, which is replaced by attS once integration has occurred. (B) Structure of SCCmec. Within SCCmec there is one direct repeat (DR) sequence, and two reverse complement inverted repeats (IRs). An additional DR is present at the boundary of the downstream chromosomal region. Arrows indicate the orientation and positions of these repeats. Sequences based on the well-characterised clinical strain of S. aureus JKD6159 (NCBI Reference Sequence: NC_017338.2).


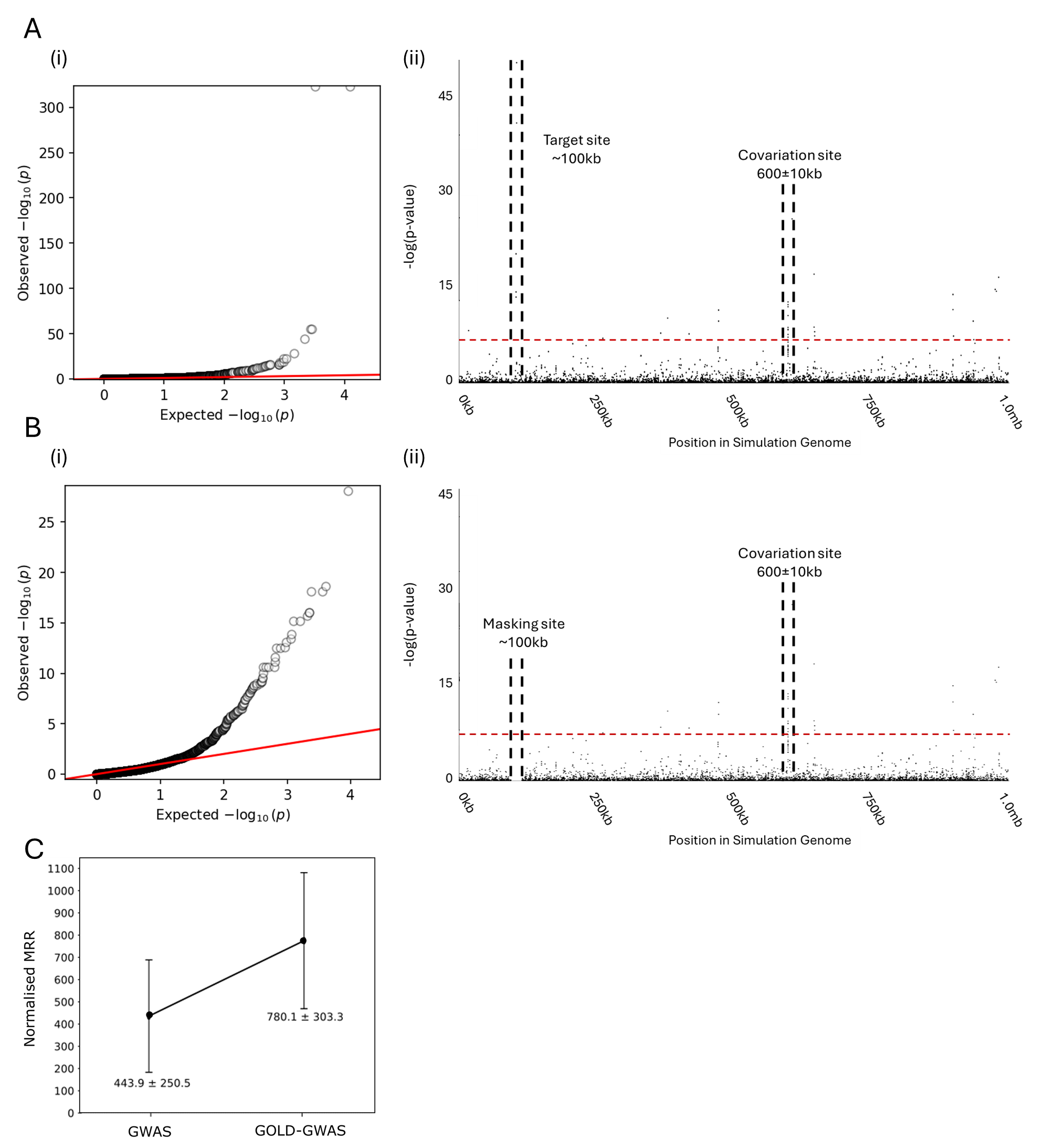


**Fig. S2: Summary of the genome-wide association study after masking an artificially created site of covariation.** (A) Standard GWAS. (i) Quantile-quantile plot comparing the expected -log(p-values) with observed -log(p-values). The red diagonal line indicates where the expected and observed values are equal. Values <10^-300^ are not shown. (ii) Manhattan plot demonstrating the statistical significance association for selected variants arranged in order on a simulated genome. Each dot represents a k-mer. Red dotted line indicates the threshold for significance. Target site near 100kb and artificial covariation region at 600kb are labelled. (B) GOLD-GWAS. (i) Quantile-quantile plot comparing the expected -log(p-values) with observed -log(p-values). The red diagonal line indicates where the expected and observed values are equal. (ii) Manhattan plot demonstrating the statistical significance association for selected variants arranged in order on a simulated genome. Each dot represents a k-mer. Red dotted line indicates the threshold for significance. Masking site near 100kb and artificial covariation region at 600kb are labelled. (C) Line plot comparing the mean reciprocal rank (MRR) of kmers for GWAS and GOLD-GWAS. Error bars represent the standard deviation.


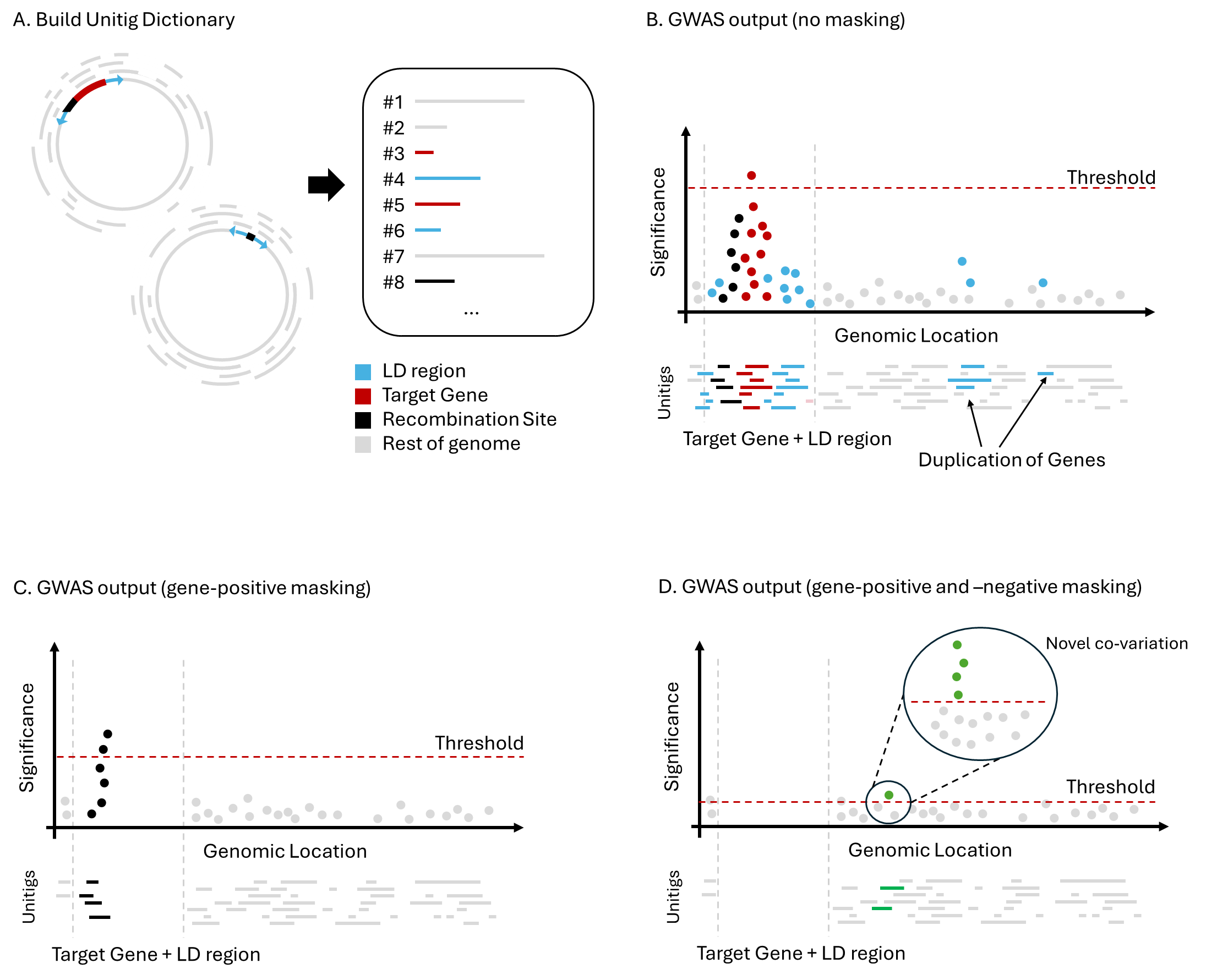


**Fig. S3: Summary of the GOLD-GWAS process.** An overview conceptualising the methodology and advantages of GOLD-GWAS over standard GWAS. (A) A dictionary of unitigs is constructed that captures all genomic variation present in a pangenome. (B) Performing standard GWAS on this unitig dictionary using the presence of a target gene as the binary classifier will produce strong correlations for any unitigs that map to the target gene itself. Consequently, the extremely high p-values associated with these unitigs will result in a high threshold for statistical significance. Strongly associated unitigs may also include a recombination site, the target gene’s linkage disequilibrium regions, and any target gene unitig duplicated within the genome. (C) After identifying all target gene and LD region unitigs within gene-positive isolates, GOLD-GWAS removes these unitigs from the dictionary before performing GWAS. Unitig filtering now allows detection of recombination site or insertion sequence due to strong correlation with gene-negative isolates only. (D) By identifying all target gene and LD region unitigs in both gene-positive and -negative isolates and removing them prior to GWAS, GOLD-GWAS reveals previously hidden unitigs that significantly covary with the target gene.
